# Simply too much: the extent to which weight bias internalization results in a higher risk of eating disorders and psychosocial problems

**DOI:** 10.1007/s40519-021-01170-z

**Published:** 2021-04-07

**Authors:** Michaela Silvia Gmeiner, Petra Warschburger

**Affiliations:** Department of Psychology, University of Potsdam, Karl-Liebknechtstr. 24-25, 14476 Potsdam, Germany

**Keywords:** Weight bias internalization, Self-stigmatization, Children, ROC, Mental health

## Abstract

**Purpose:**

Weight bias internalization (WBI) is associated with negative health consequences such as eating disorders and psychosocial problems in children. To date, it is unknown to what extent WBI considerably raises the risk of negative outcomes.

**Methods:**

Analyses are based on cross-sectional data of 1,061 children (9–13 years, *M* = 11, *SD* = 0.9; 52.1% female) who filled in the WBI scale (WBIS-C). First, ROC analyses were run to identify critical cut-off values of WBI (WBIS-C score) that identify those who are at higher risk for psychosocial problems or eating disorder symptoms (as reported by parents). Second, it was examined whether WBI is more sensitive than the relative weight status in that respect. Third, to confirm that the cut-off value is also accompanied by higher psychological strain, high- and low-risk groups were compared in terms of their self-reported depressive symptoms, anxious symptoms, body dissatisfaction, and self-esteem.

**Results:**

WBIS-C scores ≥ 1.55 were associated with a higher risk of disturbed eating behavior; for psychosocial problems, no cut-off score reached adequate sensitivity and specificity. Compared to relative weight status, WBI was better suited to detect disturbed eating behavior. Children with a WBIS-C score ≥ 1.55 also reported higher scores for both depressive and anxious symptoms, higher body dissatisfaction, and lower self-esteem.

**Conclusion:**

The WBIS-C is suitable for identifying risk groups, and even low levels of WBI are accompanied by adverse mental health. Therefore, WBI is, beyond weight status, an important risk factor that should be considered in prevention and intervention.

**Level of evidence:**

Level III, cross-sectional analyses based on data taken from a well-designed, prospective cohort study.

Weight-related stigma, i.e. associating negative stereotypes (e.g., laziness, incompetence or low willpower) with people of excess weight, is a wide-spread phenomenon [1]. It is often accompanied by discrimination and diminishes the physical and mental health of those affected [1–3]. Weight stigma can be internalized, meaning that negative attributes are accepted and applied to the self [4, 5]. This weight bias internalization (WBI) seems to be even more harmful than the mere experience of enacted discrimination [6]. Negative mental health effects of WBI matter even among children. WBI is associated with emotional problems (depressive symptoms, anxiety), reduced self-esteem, disturbed eating behavior (restrained eating, binge eating) and body dissatisfaction [6–9]. Although it is known that even children internalize weight bias, it remains unclear to what extent WBI is detrimental to mental health. Is there a critical point that indicates a higher risk of negative consequences? Especially among children, it is important to identify groups that are at higher risk—particularly in view of the fact that an early onset of mental health problems is related to a higher risk of an adverse mental health trajectory [10]. So far, only one study among an adult sample identified a critical point of WBI that goes along with depressive symptoms at a clinical level [11]. For children, comparable data are lacking. Therefore, our study aimed to identify a critical cut-off point that enables the identification of children who are at higher risk for clinically relevant disturbed eating behaviors and psychosocial problems. We hypothesized that there is a critical value of WBI that is accompanied by a higher risk of disturbed eating behavior or psychosocial problems.

People of higher weight are also at higher risk for WBI [7, 9, 12, 13], disturbed eating behaviors and psychosocial problems (e.g., depressive symptoms) [14, 15]. In any case, considering only weight status as a predictor might hinder the identification of at-risk individuals, because WBI is also present in people with normal weight [12, 16, 17]. Therefore, our second study aim was to examine whether WBI is more appropriate to identify risk groups than weight status alone.

In addition, we wanted to further validate the empirically determined cut-off value. We assumed that the low- and high-risk groups would also differ with respect to the extent of their depressive symptoms, anxiety, self-esteem, and body dissatisfaction.

## Methods

### Procedure

Data were obtained from the third measurement wave (in 2015) of the prospective PIER study, which explores intrapersonal developmental risk factors in childhood. Children from 110 elementary schools in Germany (Brandenburg) with various socioeconomic backgrounds voluntarily took part—on the condition of obtaining their parents’ informed and written consent. Children were asked to attend assessment sessions (approximately 50 min) either in quiet rooms at school or at home. Parents were invited to complete (online or paper version) questionnaires at home. Incentives in the form of small presents (such as buttons or candy) and (book) vouchers were supplied. The study was approved by the local ethics committee.

### Sample characteristics

The final sample was composed of 1,061 cases (157 cases were excluded due to missing data regarding the main outcome WBI; 442 cases were excluded because parents’ reports were lacking). Children were 9–13 years old (*M* = 11, *SD* = 0.9), 52.1% were female. In terms of weight status, 8% were classified as underweight, 78.3% as normal weight, 8.1% as overweight and 5.6% as obese [18]. Educational background was reported by the parents: 48.3% reported a higher education degree (e.g., BA, MA, Diploma, PhD etc.); 19.7% reached higher education entrance qualifications (‘Abitur’, equivalent of a high school degree/A-level) and 29.4% reported secondary school graduation or below; 2.6% did not give information about their education level.

## Materials and measures

### Sociodemographic and anthropometric data

Parental report of their highest education levels provided information about educational background. Children’s height and weight were measured by trained study personnel with calibrated instruments. Body mass index standard deviation scores (BMI-SDS) were calculated based on national reference data [18]. Gender and age were documented by the study personnel.

### Weight bias internalization

The modified Weight Bias Internalization Scale for Children (WBIS-C) [12] consists of ten items that were rated by the children on a 4-point rating scale (1 ‘*I disagree’*-4 *‘I agree’*). The scale asks participants how they apply weight-related stigma to themselves (e.g., “Because of my weight I don’t deserve to have a lot of friends and fun”). Higher scores reflect a higher internalization of weight-related stigma. Psychometric properties, reliability (α = 0.86) and factorial as well as convergent validity were satisfying [12]. Cronbach’s alpha in the current sample was α = 0.84.

### Disordered eating behavior

The SCOFF questionnaire [19] is a valid and economic screening tool to detect clinically relevant symptoms of anorectic and bulimic eating disorders by five dichotomous items (1 ‘*yes*’/ 0 ‘*no*’). It is also valid and applicable for children from an age of 12 onwards [20]. Since our children were slightly younger and pilot testing revealed comprehension problems of some items, we decided to include parental report as well. Accordingly, three items were collected via children’s self-report (losing control when eating; inducement of vomiting; believing to be too fat). It was assumed that these items can only be answered by the children themselves because the questions relate to inner regulatory processes or behavior concealed from parents. The research assistants validated the understanding of each question. The remaining two items (considerable weight loss; domination of life by food) were completed by the parents. We assumed that children would have problems rating these items because they are relatively complex and require a comprehensive judgement over time. Children’s eating behavior was classified as conspicuous if at least two items were answered in the affirmative [20]. Exploratory analyses supported this approach: children classified as conspicuous showed higher values of self-reported restrictive eating (measured by the Dutch Eating Behavior Questionnaire [21]; *t*(942) = 7.44, *p* < 0.001, *d* = 0.9) and binge eating (measured by items retrieved from the Questionnaire on Eating and Weight Patterns [22]; *t*(942) = 12.41, *p* < 0.001, *d* = 1.5). Internal consistency is not reported because the SCOFF is screens for various features that have not to be imperatively interconnected.

### Psychosocial problems

The parent version of the Strength and Difficulties Questionnaire (SDQ) [23] was used to screen for the occurrence of clinically relevant psychological problems. It is a broadly validated instrument with satisfactory psychometric properties [24] and representative norms [25]. Parents were asked to rate the occurrence of psychosocial problems (0 ‘not true’–2 ‘certainly true’) on five subscales (emotional problems, e.g., “Many worries, often seems worried”; conduct problems, e.g., “Often has temper tantrums or hot tempers”; problems with peers, e.g., “Rather solitary, tends to play alone”; hyperactivity/inattention, e.g., “Constantly fidgeting or squirming”; five items per scale). A total sum score (possible range 0–20) was calculated, with higher values indicating more problems. According to age- and gender-specific reference data [25], children above the 90th percentile were classified as showing conspicuous problems. Cronbach’s alpha of the total scale was α = 0.84.

### Additional psychosocial variables

Body dissatisfaction was measured by body silhouettes [26]. Children were presented seven (male or female) drawn body silhouettes (ranging from a very lean girl/boy to a girl/boy of excess weight). They were asked to select the figure a) that resembles them most and b) that they wish they looked like. Based on these ratings, a difference score was calculated, with higher values indicating higher body dissatisfaction. This measure was shown to be reliable and valid for children [27].

Self-esteem was assessed with the subscale of the Child Health Questionnaire (CHQ) (Landgraf et al. 1998). The CHQ showed good reliability and validity for children across several cultures (Landgraf et al. 1998). Children were asked to rate their satisfaction with different areas of life (six items, e.g., “How content are you with your performance at school?”) on a 4-point rating scale (4 ‘*very content*’–1 ‘*not content at all*’; reverse coded). Higher values represented higher self-esteem. Internal consistency was α = 0.81.

Children reported depressive symptoms based on six items taken from the German Depression Test for Children (DTGA) [28]. Depressive feelings, thoughts or behaviors (e.g., “I am often sad”) were evaluated on a 4-point rating scale (1 ‘*false*’–4 ‘*true*’). Higher mean values represented higher levels of depressive symptoms. The original scale showed acceptable reliability and validity [28]. Internal consistency in the current sample was α = 0.57.

Anxious symptoms were assessed by six items of the German Anxiety Test for Children (KAT) [29]. The items (e.g., “I often experience fear”) were rated on a 4-point rating scale (1 ‘*false*’–4 ‘*true*’). Higher scores represented higher levels of anxiety symptoms. The original scale was shown to be reliable and valid for children [29]. In this sample, internal consistency was α = 0.79.

## Analyses

### Data preparation

Overall, less than 5% of values were missing (0.46%). According to current recommendations, we used the EM algorithm [30, 31] to impute missing data.

### Identification of a WBIS-C cut-off value: ROC analyses

To examine whether WBI enables the identification of clinically relevant groups, receiving operating characteristic (ROC) curve [32, 33] analyses were run. Two ROC curves were conducted separately for disordered eating behavior and psychosocial problems. ROC curves visualize diagnostic performance by plotting the sensitivity in relation to the false-positive rate (1—specificity) [34]. To evaluate the discriminative value of the WBIS-C score, the area under the curve (AUC) was considered. It represents the accuracy and should significantly differ from 0.5 (which means that prediction is not better than random assignment). Confidence intervals (CI) are used to evaluate significance (0.5 should not be included). To attain satisfactory predictions of classification, the AUC should be at least greater than 0.7 [33, 35]. To depict the test quality, sensitivity (ability of the WBIS-C to identify those with clinically relevant problems; true positive rate) and specificity (ability to identify those without clinically relevant problems; true negative rate) are reported. The reciprocal of specificity (1—specificity) describes the rate of false alarms (participants without problems who are misclassified as having problems). To identify the WBIS-C cut-off value that best identifies groups that are at higher risk, the Youden Index was calculated [36]. According to this, the optimal value displays the maximal sum of sensitivity plus specificity.

### Comparison of the AUC of WBI and BMI-SDS

To analyze the superiority of the WBI compared to relative weight status, the AUC of the WBIS-C score was compared to the AUC of the BMI-SDS. WBI can be considered as more accurate in its predictive value in cases where the AUC of the WBIS-C score is greater than that for the BMI-SDS, and the difference between the two AUC scores (ΔAUC) is significantly different from zero (CI should not include zero).

### Validation of the cut-off value

Based on the resulting cut-off, the sample was divided into two groups with low vs. high risk. To determine the differences between these groups, they were compared with regard to additional psychosocial variables. Therefore, we performed univariate analysis of variance (ANOVAs) with WBIS-C groups (high vs. low risk) as the independent variable and the mean scores of depressive and anxious symptoms, self-esteem and body dissatisfaction as dependent variables. We observed a violation of the assumption of homogeneity of variances (indicated by Levene’s test and visual scatterplot of standardized residuals against standardized predicted values). For this reason, *F* ratios were corrected according to Welch [37]. Effect sizes (Cohen’s *d*) are interpreted according to Cohen [38]. All analyses were conducted with SPSS 26.

## Results

### Descriptive data

The mean WBIS-C Score was 1.55 (*SD* = 0.55). WBI was higher among participants with overweight or obesity (*M* = 2.09, *SD* = 0.66) compared to those of lower weight (*M* = 1.46, *SD* = 0.47; *t*(1059) = -13.9, *p* < 0.001, *d* = 1.24). Overall, 7.6% of the sample showed disturbed eating behavior (SCOFF; 21.4% in the group with overweight/obesity; 5.5% in the group with under- or normal weight). Conspicuous psychosocial problems were observed in 6% of the children (SDQ > 90th percentile; 8.3% in the group with overweight/obesity; 5.7% in the group with under- or normal weight).

### Identification of a WBIS-C cut-off value: ROC analyses

#### Disturbed eating behavior (SCOFF)

**Fig. 1:**
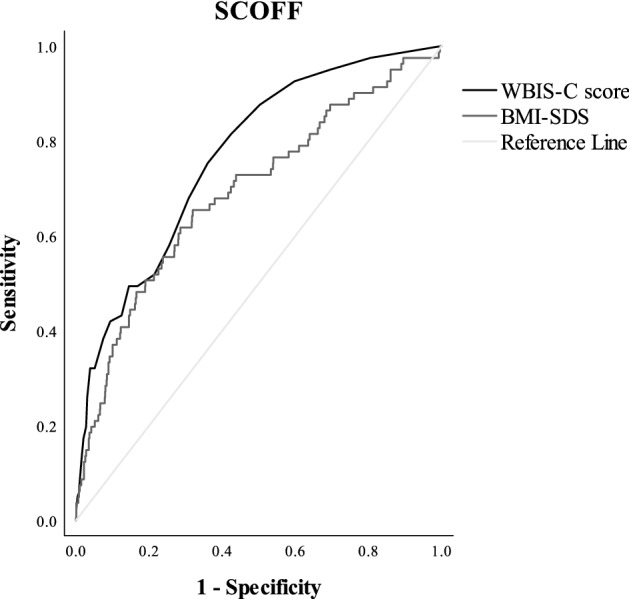
*ROC curve for disturbed eating behavior (SCOFF).* This figure illustrates the performance of WBIS-C scores to detect disordered eating behavior

The graphical ROC curve with respect to disturbed eating behavior is displayed in Fig. 1. Disturbed eating behavior can reliably be predicted by WBIS-C scores with an AUC reaching 0.77 (CI [0.72; 82]; *p* < 0.001).

The optimal cut-off point based on the Youden Index was 1.55, with a sensitivity of 0.75 (meaning that 75% of those with eating disorder symptoms were correctly classified by the WBIS-C score as having a higher risk for eating disorder symptoms) and a specificity of 0.64 (meaning that 64% of those without symptoms of eating disorders were correctly classified as non-symptomatic by the WBIS-C score).

#### Psychosocial problems (SDQ)

**Fig. 2:**
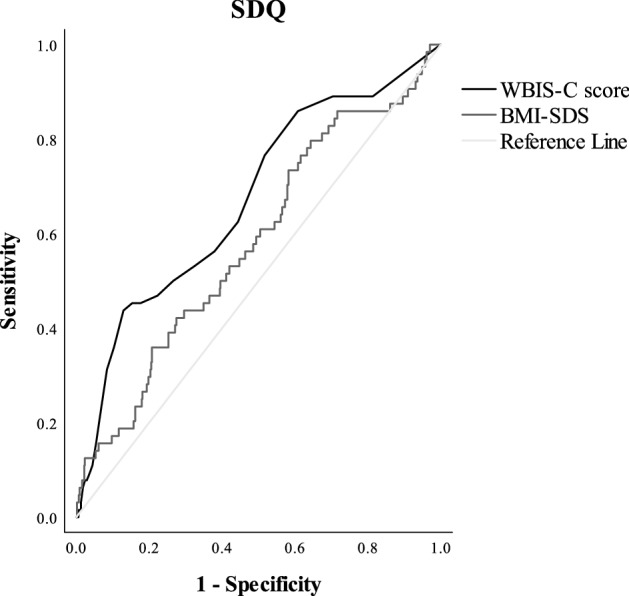
*ROC curve for psychosocial problems (SDQ).* This figure illustrates the performance of WBIS-C scores to detect psychosocial problems

The graphical ROC curve illustrating the performance of WBIS-C scores to detect psychosocial problems is depicted in Fig. 2. WBIS-C scores significantly predicted psychosocial problems (AUC = 0.67, CI [0.6; 0.74]; *p* < 0.001), but the magnitude of the AUC was not satisfying. The optimal cut-off value based on the Youden Index was 2.15, with a sensitivity of 0.44 and a specificity of 0.87.

#### Comparison of the AUC of WBI and BMI-SDS

Figures 1 and 2 also display the predictive value of BMI-SDS for disturbed eating behavior (AUC = 0.69, CI [0.63; 0.76], *p* < 0.001) and psychosocial problems (AUC = 0.58, CI [0.51; 0.66], *p* = 0.032), respectively. To answer the question whether the WBIS-C scores are better able to predict disturbed eating disorders than the BMI-SDS, the corresponding AUCs were compared. The ΔAUC scores revealed that the performance of WBIS-C scores is more accurate than BMI-SDS for both disturbed eating behavior (ΔAUC = -0.08; CI [-0.13; -0.02], *p* = 0.006) and psychosocial problems (ΔAUC = -0.09; CI [-0.17; -0.01], *p* = 0.021).

#### Validation of the cut-off value

As the results with respect to the SDQ values were not sufficient, risk groups were formed based on the WBIS-C cut-off point for the SCOFF. According to this cut-off point, 39% of children were allocated to the high-risk group.

**Fig. 3:**
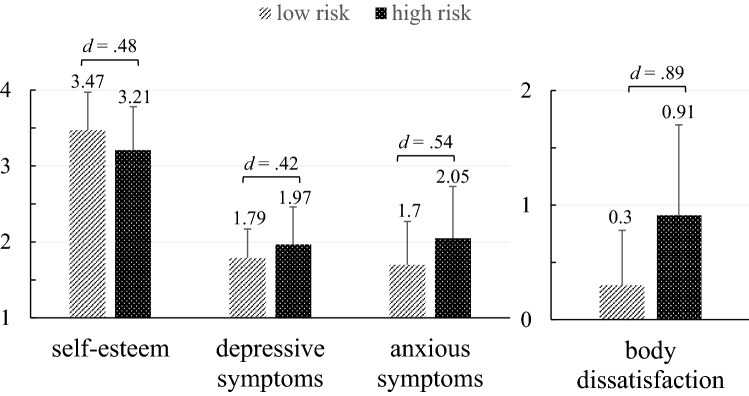
*Self-esteem, depressive symptoms, anxious symptoms and body dissatisfaction: comparison of the WBIS-C high- and low-risk groups.* This figure displays the mean scores for the low- (*n* = 647) versus high-risk group (*n* = 414). All differences were statistically significant (*p* < 0.001). The vertical lines display the standard deviation of the mean scores. Effect sizes are displayed as Cohen’s *d*

Figure 3 displays the mean scores of the additional psychosocial variables for the high versus low-risk group. We observed medium to large effect sizes for depressive symptoms (*F*(1, 724.34) = 44.65, *p* < 0.001), anxious symptoms (*F*(1, 773.36) = 74.36, *p* < 0.001), self-esteem (*F*(1, 802.12) = 57.36, *p* < 0.001) and body dissatisfaction (*F*(1, 612.64) = 201.97, *p* < 0.001).

## Discussion

WBI is associated with detrimental effects on health, but it remains unclear to what extent WBI considerably increases the risk for negative outcomes among children. The results underscore that even relatively low levels of WBI (WBIS-C cut-off value 1.55) indicate a heightened risk for pathological eating behaviors. It has also been shown that WBI, compared to BMI-SDS, is better suited to identifying a high-risk group. This high-risk group also exhibited higher depressive and anxious symptoms, higher body dissatisfaction and lower self-esteem compared to the low-risk group.

To the best of our knowledge, this is the first study that examines a critical threshold of WBI in children. There is only one other study [11] examining cut-off points for WBI among adult patients with overweight or obesity. The authors reported an acceptable AUC, sensitivity and specificity for their cut-off point, and concluded that their Italian WBI scale is suited to identifying different levels of depression. Contrary to their results, the application of the WBIS-C to identify psychosocial problems in children cannot be recommended. Although a similar cut-off value (comparison based on standardized values) for psychosocial problems (SDQ) was identified, the psychometric parameters in the present study were not acceptable. It should be taken into account that Innamorati et al. [11] referred to a treatment-seeking sample with a higher prevalence of psychological strain [39]. In the current sample, the occurrence of conspicuous psychological problems among children was relatively seldom, albeit representative for Germany [40]. These diverse findings indicate that it might be interesting to examine whether the WBIS-C better detects psychosocial problems within clinical settings.

With regard to disturbed eating behavior, the data showed that WBI is appropriate for identifying those who are at risk. Hereby, the sensitivity is greater than the specificity. The higher sensitivity seems to be acceptable because it is more important to detect those who are probably at risk, whereas it is more detrimental to miss those who are at risk. The mean WBIS-C score in the present sample and the identified cut-off value are relatively low (below the midpoint of the scaling, which would be 2.5) indicating that WBI averages out at a relatively low level. However, even low levels of WBI were shown to be accompanied by a higher risk for eating disorder symptoms and higher depressive symptoms, anxiety symptoms, body dissatisfaction and reduced self-esteem as well.

To sum up, the present study highlights that even low levels of WBI in children might be associated with adverse mental health. Hereby, WBI seems to be an even better predictor than relative weight status alone. On the one hand, this means that targeting WBI within the framework of broad prevention strategies, e.g., at schools, could be promising. On the other hand, the cut-off value could be administered in clinical settings to identify those who are at risk and psychosocial support should be offered. Either way, as WBI is associated with several negative outcomes, already low levels of WBI require attention and call for respective interventions. For example, promoting body positivity might be a suitable anti-stigmatizing approach, as it addresses body acceptance and appreciation as well as appearance ideals [41, 42].

## Strengths, limitations, and future implications

The present study reveals that it is important to consider WBI in children, as even low levels of WBI are associated with impaired mental health. Nevertheless, some limitations should be considered: first, it has to be mentioned that the data do not refer to clinical diagnoses. This is due to practical and economic reasons as part of the overall study design. Instead, established questionnaires with broadly validated cut-off values to identify those with eating disorder symptoms or psychosocial problems of clinical relevance were applied. However, the procedure of combining child and parent report to assess conspicuous eating behavior was not validated with an independent sample. Hence, the results should be supported by a further validation including clinical interviews or diagnoses. Second, the operationalization of eating disorder symptoms and psychosocial problems was mainly based on parental report. This seems to be adequate for this age group, but internalizing psychosocial problems are probably underestimated [43, 44]. Third, the results are limited by the cross-sectional design. The current data allow no interpretation with respect to the temporal relationship of the variables. Besides internalization being associated with impaired mental health, it is reported that poor psychosocial health or eating pathology might in turn increase vulnerability of internalization [45]. Prospective longitudinal research is needed to verify the predictive value of WBI over the long term and to examine potential bidirectional relationships.

It should also be mentioned that the shortened scale of the DTGA showed low reliability. Therefore, the results with respect to depressive symptoms should be interpreted with caution and confirmed in further studies. Fourth, the sample was split into a high- and a low-risk group to further validate the cut-off score. This might lead to a comparison of lopsided groups (as the cut-off score that was generated within the same sample; see [46]). Therefore, the results are preliminary and have to be interpreted with caution. Last but not least, the generalization of the present results might be partly restricted as the sample mainly consisted of children from families with an educational level above average. Previous analyses showed that a lower educational level is a risk factor for higher WBI [45], which might result in underestimating the relevance of WBI in the present analyses.

The current study also has several strengths. The data refer to children—a vulnerable life stage, particularly with respect to the possible detrimental effects of WBI [2]. An early onset of these mental problems in childhood is in turn connected with stable adverse developmental pathways [10]. Thus, the data that trace back to a non-clinical sample allow identifying children who are at risk, probably also before they meet the clinical threshold for an eating pathology, and provides the opportunity to prevent adverse mental health trajectories. Further, the WBI cut-off value was validated with additional assessments. This showed that those who are identified as being at high risk actually also reported higher psychological strain. Additionally, the present study included a large sample with an equal number of girls and boys across different weight groups representative for Germany [40]. As previously recommended, WBI was measured with items that refer to weight in general and not solely overweight/obesity [47]. This allowed to assess WBI across different weight categories and extend previous research that has often focused on overweight [6, 9, 11]. To sum up, the findings underscore that WBI is even better suited to identify those of higher risk than the relative weight status.

## Conclusion

Overall, the results suggest that the WBIS-C is a suitable tool to identify risk groups with suspicious WBI. As even low WBI levels are accompanied by adverse mental health, WBI requires attention among children and adolescents across all weight groups. The WBIS-C enables an early detection of vulnerable groups in the general population as well as within clinical samples. The results show that it is important to promote appropriate prevention strategies to reduce weight bias internalization in the general population (e.g., at schools) as well to consider WBI in intervention strategies within clinical settings (e.g., in the context of eating disorders).

## What is already known on this subject?

Weight bias internalization (WBI) is associated with increased mental health problems in children and adolescents. The extent to which WBI considerably raises the risk of negative outcomes is unknown.

## What this study adds?

The WBI scale for children is suitable for identifying risk groups; even low WBI levels are accompanied by adverse mental health. WBI should be considered in prevention and intervention.

## Data Availability

The datasets generated and analyzed during the current study are not publicly available, as the participants were not asked to consent to publication within repositories, but are available from the corresponding author on reasonable request.
